# Why calpain inhibitors are interesting leading compounds to search for new therapeutic options to treat leishmaniasis?

**DOI:** 10.1017/S003118201600189X

**Published:** 2016-11-21

**Authors:** VITOR ENNES-VIDAL, RUBEM FIGUEIREDO SADOCK MENNA-BARRETO, MARTA HELENA BRANQUINHA, ANDRÉ LUIS SOUZA DOS SANTOS, CLAUDIA MASINI D'AVILA-LEVY

**Affiliations:** 1 Laboratório de Estudos Integrados em Protozoologia, Instituto Oswaldo Cruz/FIOCRUZ, Rio de Janeiro, Brazil; 2 Laboratório de Biologia Celular, Instituto Oswaldo Cruz/FIOCRUZ, Rio de Janeiro, Brazil; 3 Laboratório de Investigação de Peptidases, Universidade Federal do Rio de Janeiro, Rio de Janeiro, Brazil

**Keywords:** Calpain inhibitors, leishmaniasis, repurpose approach, MDL28170

## Abstract

Leishmaniasis is a neglected disease, which needs improvements in drug development, mainly due to the toxicity, parasite resistance and low compliance of patients to treatment. Therefore, the development of new chemotherapeutic compounds is an urgent need. This opinion article will briefly highlight the feasible use of calpain inhibitors as leading compounds to search for new therapeutic options to treat leishmaniasis. The milestone of this approach is to take advantage on the myriad of inhibitors developed against calpains, some of which are in advanced clinical trials. The deregulated activity of these enzymes is associated with several pathologies, such as strokes, diabetes and Parkinson's disease, to name a few. In *Leishmania*, calpain upregulation has been associated to drug resistance and virulence. Whereas the difficulties in developing new drugs for neglected diseases are more economical than biotechnological, repurposing approach with compounds already approved for clinical use by the regulatory agencies can be an interesting shortcut to a successful chemotherapeutic treatment for leishmaniasis.

## INTRODUCTION

The Trypanosomatidae family, Kinetoplastea class, is composed of a large group of exclusively parasitic protozoa, some of which cause important diseases in humans (d'Avila-Levy *et al.* 2015). Altogether, about 37 million people worldwide are infected with *Trypanosoma brucei*, the aetiological agent of African sleeping sickness; *Trypanosoma cruzi*, the causative agent of Chagas’ disease or American trypanosomiasis; and different species of the genus *Leishmania*, which are responsible for a wide spectrum of clinical manifestations known as cutaneous, mucocutaneous and visceral leishmaniasis (WHO, 2015). Since these diseases have been wiped out in the more developed parts of the world and persist only in the poorest, most marginalized communities and conflict areas, they are classified as neglected tropical diseases by the World Health Organization (WHO, 2015). Factors like malnutrition, weak immunity, illiteracy, lack of resources and environmental changes, as well as the migration of non-immune people to endemic areas, play important roles in the dynamic of these diseases (Desjeux, 2001; Alvar *et al.* 2006; Boelaert *et al.* 2010).

All the clinical manifestations collectively known as leishmaniasis comprise one of the most prevalent neglected diseases worldwide with more than 2 million new cases occurring annually and endangering around 350 million people in 98 countries in the tropics, sub-tropics and European Mediterranean area (WHO, 2015). Visceral leishmaniasis, also known as kala-azar, is the most severe one, and can be fatal if untreated. This clinical manifestation affects the vital organs of the body and is characterized by irregular bouts of fever, weight loss, anaemia, and swelling of the spleen and liver. If not fully healed, visceral leishmaniasis can progress to post-kalazar dermal leishmaniasis, which is characterized by a hypopigmented macular, maculopapular and nodular rash. Cutaneous leishmaniasis, the most common form of the disease, causes ulcers on exposed skin areas of the body, leading to disfigurement, permanent scars, social stigma and in some cases disability. However, this is usually a self-healing illness. Finally, mucocutaneous leishmaniasis consists of the most destructive form of the disease, since it causes partial or total mutilation of mucous membranes in the nose, mouth and throat. In almost all cases, it may cause serious deformities (WHO, 2010; Alvar *et al.* 2012).

## TREATMENT AND DRUG THERAPY

Despite the great advances in combating infectious diseases over the past century, the current therapy to treat neglected diseases, like leishmaniasis, is extremely limited to a handful of drugs that suffer from unacceptable toxicity, high costs, difficulties of administration and increasing treatment failures, since resistance to these compounds has become a severe problem (Cavalli and Bolognesi, 2009; Wilkinson and Kelly, 2009; Boelaert *et al.* 2010). In view of this scenario, the development of new drugs is an urgent need, which has led to the investigation of several compounds chosen empirically, or through studies that identify promising metabolic targets to the rational drug design and selection (De Menezes *et al.* 2015).

**Fig. 1.**
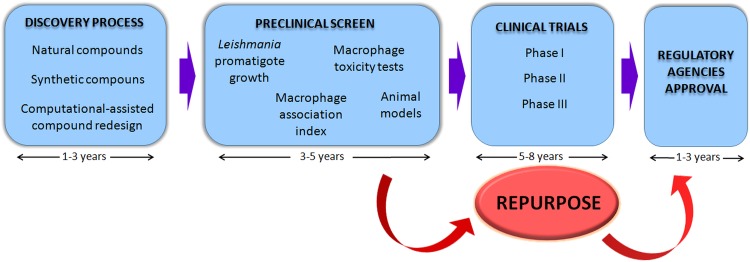
Representation of the main steps necessary to the final drug approval and the shortcut represented by the repurpose approach.

The process of drug development is time consuming, laborious and expensive. On average, a drug is developed in 15–17 years, from the discovery process to pharmacological regulatory agencies approval. Before the availability for doctors to prescribe, millions are spent, starting from the discovery process that involves screening chemically diverse compounds (synthetic or natural sources), computational-assisted compound redesign, preclinical testing in cellular and animal models, to clinical trials and then the final approval (Fig. 1). Considering the neglected diseases, the reality is quite different, since the link with poverty results in low investment in rational drug development. A high number of patents are dead due to the failure of payment of maintenance cost, or revoked for not meeting patentability requirements, which seems to be an indication of the difficulties faced by research institutes and universities for disclosed compounds to reach the final stages of innovation and entering into the market (Machado-Silva *et al.* 2015). Consequently, governmental action is crucial to guarantee innovation and patient care and treatment. Therefore, considering that the challenges for the introduction of new compounds to treat neglected diseases are more economical than biological, repurpose drugs have potential benefits, such as, reducing the costs during discovery and development, preclinical laboratory tests and clinical phases (Andrews *et al.* 2014; Kwok and Koenigbauer, 2015) (Fig. 1).

## CALPAIN INHIBITORS

**Fig. 2.**
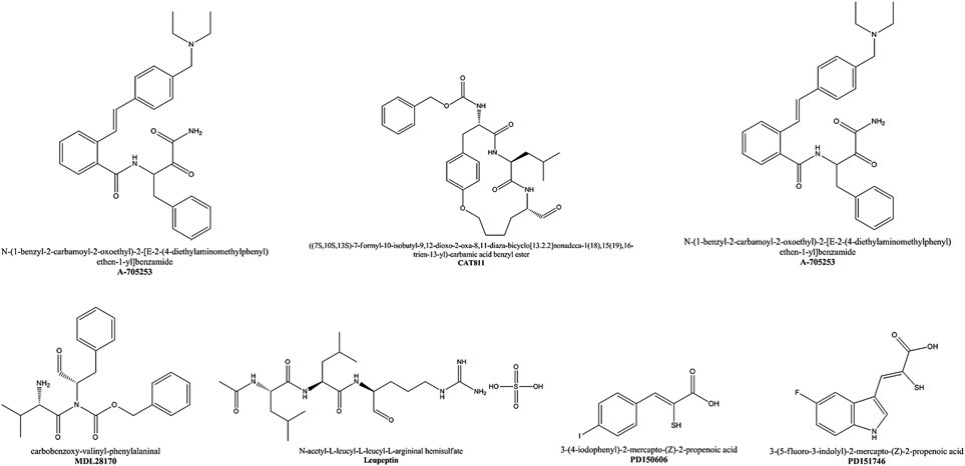
Chemical structures of selected calpain inhibitors, for an extensive list refer to Donkor (2015).

Economically, drug-repurposing strategies have the potential to facilitate an effective drug development. As the cost associated with discovering and developing a new chemical entities may be around US$ 800 million and may take one to two decades, repurpose drugs have already proven to have considerable advantages (Ashburn and Thor, 2004; Taylor and Wainwright, 2005; Nwaka and Hudson, 2006; Andrews *et al.* 2014) (Fig. 1). Taking into consideration this last possibility, a family of neutral calcium-dependent cysteine peptidases, the calpains, calls attention because a huge effort has been made to develop means of identifying selective inhibitors (Carragher, 2006; Donkor, 2015). These enzymes are involved in a variety of calcium-regulated cellular processes, such as signal transduction, cytoskeleton remodelling, cellular proliferation and differentiation, sex determination, membrane fusion, environmental regulated processes and apoptosis. Besides their physiological roles, calpains unregulated activity in humans is implicated in several pathophysiological processes, such as, aging, muscular dystrophy, multiple sclerosis, cataract, arthritis, cancer, strokes, diabetes and neurological disorders (Alzheimer's, Huntington's and Parkinson's diseases). It has been advocated that the specific inhibition of calpains under these condition can treat these pathologies. In addition to these pathologies, calpain unregulated activity also plays a crucial role in neuron death in traumatic spinal cord injury, and its specific inhibition can prevent apoptosis and restore transcription of proteolipid protein and myelin basic protein genes, which indicates the therapeutic efficacy of calpain inhibitors to rescue or prevent permanent disability. Therefore, prompt inhibition of calpains, if undertaken early enough after injury, could significantly spare many neurons (Huang and Wang, 2001; Battaglia *et al.* 2003; Saez *et al.* 2006; Zhang *et al*. 2015). To treat some of these pathologies, in the last 4 years, at least 52 calpain inhibitors were developed and screened (Donkor, 2015). Out of these, one is under phase I clinical trial to treat Alzheimer disease, and another compound presented promising results in animal models to the treatment of cataracts and clinical trials will be performed by Calpain Therapeutics Co. (Table 1; Fig. 2). Up to date, calpain inhibitors display a wide range of potency, but low specificity, inhibiting also other cysteine and serine peptidases or even the proteasome (Low *et al.* 2016). Therefore, future efforts in the development of calpain inhibitors should not only concentrate on potency and selectivity of the inhibitors for calpain compared with other proteases but should also focus on achieving significant calpain isoform selectivity (Donkor, 2011).

**Table 1.** A non-comprehensive list of available calpain inhibitors and its potentiality for clinical use, for an extensive list refer to Donkor (2015).

Inhibitors name, Alias	Most important structures	Commercial availability	Notes
A-705253, ABT-957	(*N*-(1-benzyl-2-carbamoyl-2-oxoethyl)-2-[*E*-2-(4-diethlyaminomethylphenyl) ethen-1-yl]benzamide	Abbvie Inc.	In Phase I clinical trial to treat Alzheimer`s Disease (ClinicalTrials.gov Identifier: NCT0222073)
CAT811	((7S,10S,13S)-7-Formyl-10-isobutyl-9,12-dioxo-2-oxa-8,11- diaza-bicyclo[13·2·2]nonadeca-1(18),15(19),16-trien-13- yl)-carbamic Acid Benzyl Ester	Calpain Therapeutics Pty Ltd.	Safety and efficacy established in animals models to treat cataracts, and currently undergoing confirmatory preclinical studies in human lens model to progress to clinical trials (Morton *et al.* 2013).
PD150606 and PD151746	3-(4-Iodophenyl)-2-mercapto-(Z)-2-propenoic acid, and 3-(5-Fluoro-3-indolyl)-2-mercapto-(Z)-2-propenoic acid	Merck Millipore Co.	Although high selective to calpains in preclinical studies to treat muscular disorders, analogues are under development to favour inhibition of calpain-1 over calpain-2
E-64 and derivates	[L-trans-3-Carboxyoxirane-2-carbonyl]-L-Leu-agmatine	Sigma Aldrich Co., Merck Millipore Co., Peptide Inst. and Bachem AG.	Safety and efficacy of such compounds had already been demonstrated in preclinical and (Hook *et al.* 2007) and clinical studies (Satoyashi, 1992), but strategies for enhancing the selectivity for calpain isoforms should be enhanced
Calpain Inhibitor III, MDL-28170 and analogs	Carbobenzoxy-valinyl-phenylalaninal	Merck Millipore Co., Sigma Aldrich Co., Bachem AG. and Enzo Life Sciences Inc.	A powerful and cell-permeable calpain inhibitor (Mehdi, 1991), but presents cross reactivity with cathepsins B and L. Novel highly selective analogues provided promising *in vitro* results (Kim *et al.* 2011)
Leupeptin derivates	Ac-Leu-Leu-L-Argininal	Sigma Aldrich Co., Peptide Inst. and Bachem AG	Derivates of leupeptin are under development as a means of facilitating penetration of the inhibitor into the cells (reviewed by Donkor, 2011).
BDA-410	(2S)-N-{(1S)-1-[(S)-Hydroxy(3-oxo-2-phenyl-1-cyclopropen-1-yl)methyl]-2-methylpropyl}−2-benzenesulfony-lamino-4-methylpentanamide	Mitshubichi Tanabe Pharma Co.	Relatively selective inhibitor of calpain-1 rather than calpain-2 presenting promising results in Alzheimer's disease in preclinical studies (Battaglia *et al.* 2003)

Detailed studies on the kinetics and mechanism of action of calpain inhibitors allowed to classify them as: allosteric effectors, mechanism-based, tight binding, slow binding, affinity labels, suicide substrates, transition state analogues and dead-end inhibitors (Otto and Schirmeister, 1997; Vicik *et al.* 2006). Allosteric calpain inhibitors are known not to target the active site but most likely interact with allosteric sites, which are involved in catalysis and activation; as a result, these molecules may provide more therapeutic benefit than peptide inhibitors. An example is *α*-mercaptoacrylate PD150606, a potent and selective inhibitor of calpain-1 (Wang *et al.* 1996).

Active site-directed inhibitors, as the name suggests, interact directly with the active site. These compounds are usually peptidomimetics of calpain substrates, composed of modified amino acids that are recognized by the enzyme, and can be either reversible or irreversible inhibitors (Angelastro *et al.* 1990). Examples of the former include the synthetic compounds aldehydes, *α*-ketoheterocycles, and *α*-ketocarbonyls (Kawasaki *et al.* 1989; Tao *et al.* 1998), and natural compounds such as leupeptin (Mehdi 1991). Unfortunately, these inhibitors lack specificity, and attempt to improve them lead to loss of potency; additional problems to be surpassed are membrane permeability and solubility (Low *et al.* 2016).

Calpastatin is the natural endogenous inhibitor of calpains, and it is highly specific; its specificity is determined by the simultaneous binding of three calpastatin subdomains to both subunits of heterodimeric calpains (Battaglia *et al.* 2003; Carragher, 2006). Considering that the entire protein poorly penetrates the cells, fusion proteins containing a calpastatin peptide and a signal sequence capable of delivering the fusion protein into the cells have been produced and patented (reviewed by Donkor, 2015). Also, taking into consideration the mode of action of calpastatin, i.e. a β-turn loop within calpastatin forms a broad interaction around the active site cysteine that inhibits the enzyme, a library of peptidic compounds was constructed and are under test (Low *et al.* 2016). There are some promising results on Ki with a 1000-fold selectivity for calpain compared with cathepsin L (Jiao *et al*. 2010).

## CALPAINS IN TRYPANOSOMATIDS

Although calpains are well described in mammalian cells as well as its physiological roles and involvement in pathological disorders, in trypanosomatids, the picture is different. Calpains have a wide variety of domains in addition to the peptidase domain, such as calcium binding and penta-EF-hand domains. The calpain superfamily is divided into several subfamilies according to the structures of these additional domains. Since the mammalian conventional calpain catalytic subunits are the reference point for calpain structure, calpains having a similar domain structure are called ‘classical’ calpains in contrast to ‘non-classical’ ones that may lack one or all of these domains, including the peptidase domain. Non-classical calpain peptidase domains have amino acid identities with each other ranging from<25% to >75%, and they may have other functional domains in aleatory regions of the protein (Sorimachi *et al.* 2011). In addition, several of these non-classical calpains have alterations in the catalytic triad leading to proteins devoid of proteolytic activity.

A survey on trypanosomatids genome revealed a total of 18 calpain-like sequences in *T. brucei*, 24 in *T. cruzi* and 27 in *Leishmania major* (Ersfeld *et al.* 2005). Among these, some proteins present the catalytic triad conserved, which supports the idea that calpains may have proteolytic activity in trypanosomatids. However, these enzymes are tricky to detect biochemically, and may be readily hydrolyzed by other abundant peptidases. In this sense, calpain proteolytic activity was never demonstrated in *T. cruzi* or *Leishmania* spp. However, in *Angomonas deanei* (formerly *Crithidia deanei*), a monoxenic trypanosomatid, a proteolytically active cysteine peptidase was purified and presented several biochemical characteristics of calpains, such as neutral pH and loss of activity upon ions chelation, which was recovered after calcium restoration. Although the amino acid sequence of the purified protein was not demonstrated, the protein cross reacted with antibodies raised against an atypical calpain from *Drosophila mellanogaster* (d'Avila-Levy *et al.* 2003). Also, two reports suggest the presence of a calcium-dependent cysteine peptidase in *Leishmania donovani*, but the molecular identity of the enzyme was also not assessed (Bhattacharya, Dey & Datta 1993; Dey *et al.* 2006). Therefore, more efforts should be directed to ascertain whether calpains are proteolytically active in trypanosomatids.

Considering the high number of genes and sequence diversity of calpains in trypanosomatids, it is not an easy task to completely characterize this protein family and to assess its functions. Up to now, there is no knockout available. However, in *T. brucei*, the RNAi of three calpain genes revealed their roles in parasite growth, morphology and flagellum assembly (Olego-Fernandez *et al.* 2009; Hayes *et al.* 2014).

There are several other evidences on the relevance of calpain molecules for *Leishmania* life cycle disclosed by unbiased assays, such as transcriptomics and proteomics approaches (reviewed by Branquinha *et al*. 2013). When highly sensitive gene expression microarray technology was employed to identify genes that are differentially expressed in *L. donovani* isolated from post kala-azar dermal leishmaniasis (PKDL) patients in comparison with those from visceral leishmaniasis, a 2-fold higher expression of five proteins in PKDL parasites was reported, including a short calpain (Salotra *et al.* 2006). In another approach, a comparative proteomics screen between antimonial-resistant and -sensitive *L. donovani* strains isolated from kala-azar patients revealed a calpain-related protein SKCRP14·1, which is downregulated in the resistant strain, and modulate the susceptibility to antimonials and miltefosine by interfering with drug-induced programmed cell death (PCD) pathways: when over-expressed, this calpain significantly increased the sensitivity of the resistant strain to antimonials, being able to promote PCD, but the opposite effect was seen in miltefosine-treated cells, in which this calpain molecule protected against miltefosine-induced PCD. It was concluded that the calpain SKCRP14·1 is likely to be a regulator of PCD (Vergnes *et al.* 2007). As a matter of fact, conflicting roles for calpain activity in contributing to the promotion and/or suppression of apoptosis have been proposed in mammals, being suggested that calpains must have a wide influence over many apoptotic processes, and their specific roles during apoptosis may differ depending on the cell type and the nature of the apoptotic stimulus. In *L. major* differentiation from procyclic-into-metacyclic promastigotes, one calpain gene was shown to be upregulated in the procyclic promastigote insect stage, while two distinct calpains were upregulated in the metacyclic insect stage through DNA microarray analysis. Life cycle-specific expression may also demarcate the search for specific functions of these calpains (Saxena *et al*. 2003). In trypanosomatids, it is possible that the great expansion of the calpain family in the parasite genome together with the variety in calpains structure in comparison with the mammalian calpains may contribute to the variety of functions performed (Ersfeld *et al.* 2005; Branquinha *et al.* 2013).

The link between the available calpain inhibitors and a ‘me-too’ or repurpose approaches for leishmaniasis chemotherapy encouraged our research group to assess the effects of a calpain inhibitor against pathogenic trypanosomatid parasites (d'Avila-Levy *et al.* 2006; Sangenito *et al.* 2009; Ennes-Vidal *et al.* 2010, 2011; Branquinha *et al*. 2013; Marinho *et al.* 2014). MDL28170 (Cbz-Val-Phe-H) is a potent, cell-permeable, synthetic and reversible peptide inhibitor of calpain I and II, also known as calpain inhibitor III (Mehdi 1991). Up to now, this compound showed promising results in pre-clinical studies *in vitro* with some *Leishmania* species. MDL28170 interfered in various steps of the parasite life cycle and incited our research group to program further studies to better understand the calpain functions in these organisms. Our results showed that MDL28170 was capable of arresting irreversibly the growth of *L. amazonensis* promastigotes in a dose-dependent manner (d'Avila-Levy *et al.* 2006), while one possible mechanism of action is through the activation of an apoptotic-like pathway (Marinho *et al.* 2014). Unpublished results from our research group indicate that MDL28170 is also capable of arresting the development of two *Leishmania* species in host cells. MDL28170 was also effective against all the morphological stages found in *T. cruzi*, including bloodstream trypomastigote, the most infective stage of the parasite (Sangenito *et al.* 2009; Ennes-Vidal *et al.* 2010). In addition, either the treatment of *T. cruzi* with MDL28170 prior to host cell infection, or the post-infection treatment, considerably reduced infection (Ennes-Vidal *et al.* 2010). The calpain inhibitor also arrested the *in vitro* metacyclogenesis of *T. cruzi* and impaired parasite adhesion (epimastigote forms) to the gut of the insect vector *Rhodnius prolixus* in a dose-dependent manner (Ennes-Vidal *et al.* 2011). Interestingly, the compound did not display any relevant cytotoxic effect on mammalian host cells in the concentrations that caused a considerably reduction on the parasite development in the host cells. It is possible that the parasite may concentrate the inhibitor, or even that the diversity of cysteine peptidases in the host cells may surpass the functions blocked by the inhibitor, thus reinforcing the possibilities for chemotherapeutic intervention. Nevertheless, it should be pointed out that MDL28170 as other calpain inhibitors may also act on other parasite peptidases, also the micromolar concentration required for activity may reflect the low affinity to a possible calpain target or even its action on other unpredicted targets. However, its action even in the micromolar ranges encourages the pursuit calpains and calpain inhibitors as a possible chemotherapeutic approach, but with several challenges: identify, purify and characterize an active calpain; determine its structure; identify and select possible inhibitors and test these inhibitors in pre-clinical assays. Also, the new generation of calpain inhibitors should be continuously checked for anti-leishmanial activity.

### Concluding remarks

Here, we discuss the repurpose approach as a viable economic alternative to circumvent the bottlenecks of drug discovery pipeline, particularly in neglected disease. There are already examples of a blockbuster success of repurpose drug, such as the Pfizer's Viagra (Sildenafil), which is used to combat erectile dysfunction. Initially, the drug was developed for heart disease treatment, but the observation that penile erections were a common side effect in phase I trials led to pilot studies with Sildenafil in male erectile dysfunction (Ghofrani *et al.* 2006). Interestingly, in *Leishmania*, there is already an example of a repurpose drug. Miltefosine was initially described with antiprotozoal and antineoplastic, however, the application of this compound in an oral formulation in the treatment of different tumours was discontinued, and the successful trials outcomes in India led to approval of the compound for the treatment of visceral leishmaniasis (Dorlo *et al*. 2012). Therefore, a repurpose approach with approved inhibitors could be an interesting shortcut for the treatment of leishmaniasis, and considering that calpain inhibition is an explored pathway to treat strokes, diabetes and Parkinson's disease, among others, including the possible prevention of spinal cord injury and permanent disability, we advocate that calpain inhibitors should be explored as potential chemotherapeutic agents to treat leishmaniasis. Likewise, other compounds or inhibitors suitable for a repurpose approach should be explored by the scientific community. Also, the calpain family needs more efforts to better characterize it in trypanosomatids, improving not only our knowledge on this intriguing family of peptidases, but also helping in rational drug design approaches.

## References

[ref1] Alvar J., Yactayo S. and Bern C. (2006). Leishmaniasis and poverty. Trends in Parasitology 22, 552–557. 17023215 10.1016/j.pt.2006.09.004

[ref2] Alvar J., Velez I. D., Bern C., Herrero M., Desjeux P., Cano J., Jannin J. and den Boer M. (2012). Leishmaniasis worldwide and global estimates of its incidence. Plos ONE 7, e35671. 22693548 10.1371/journal.pone.0035671 PMC3365071

[ref3] Andrews K. T., Fisher G. and Skinner-Adams T. S. (2014) Drug repurposing and human parasitic protozoan diseases. International Journal for Parasitology: Drugs and Drug Resistance 4, 95–111. 25057459 10.1016/j.ijpddr.2014.02.002 PMC4095053

[ref4] Angelastro M. R., Mehdi S., Burkhart J. P., Peet N. P. and Bey P. (1990). Alpha-diketone and alpha-keto ester derivatives of N-protected amino acids and peptides as novel inhibitors of cysteine and serine proteinases. Journal of Medicinal Chemistry 33, 11–13. 2296008 10.1021/jm00163a002

[ref5] Ashburn T. T. and Thor K. B. (2004). Drug repositioning: identifying and developing new uses for existing drugs. Nature Reviews Drug Discovery 3, 673–683. 15286734 10.1038/nrd1468

[ref6] Battaglia F., Trinchese F., Liu S., Walter S., Nixon R. A. and Arancio O. (2003). Calpain inhibitors, a treatment for Alzheimer's disease: position paper. Journal of Molecular Neuroscience 20, 357–362. 14501020 10.1385/JMN:20:3:357

[ref7] Bhattacharya J., Dey R. and Datta S. C. (1993). Calcium dependent thiol protease caldonopain and its specific endogenous inhibitor *in Leishmania donovani*. Molecular and Cellular Biochemical 126, 9–16. 10.1007/BF01772203 8107694

[ref8] Boelaert M., Meheus F., Robays J. and Lutumba P. (2010). Socio-economic aspects of neglected diseases: sleeping sickness and visceral leishmaniasis. Annual Tropical Medicinal Parasitology 104, 535–542. 10.1179/136485910X12786389891641 21092391

[ref9] Branquinha M. H., Marinho F. A., Sangenito L. S., Oliveira S. S. C., Gonçalves K. C., Ennes-Vidal V., d'Avila-Levy C. M. and Santos A. L. S. (2013). Calpains: potential targets for alternative chemotherapeutic intervention against human pathogenic trypanosomatids. Current Medicinal Chemistry 20, 3174–3185. 23899207 10.2174/0929867311320250010 PMC4181241

[ref10] Carragher N. O. (2006). Calpain inhibition: a therapeutic strategy targeting multiple disease states. Current Pharmaceutical Design 12, 615–638. 16472152 10.2174/138161206775474314

[ref11] Cavalli A. and Bolognesi M. L. (2009). Neglected tropical diseases: multitarget-directed ligands in the search for novel lead candidates against *Trypanosoma* and *Leishmania*. Journal of Medicinal Chemistry 52, 7339–7359. 19606868 10.1021/jm9004835

[ref12] d'Avila-Levy C. M., Souza R. F., Gomes R. C., Vermelho A. B. and Branquinha M. H. (2003). A novel extracellular cysteine proteinase from *Crithidia deanei*. Archives of Biochemical and Biophysical, 420, 1–8. 10.1016/j.abb.2003.09.033 14622969

[ref13] d'Avila-Levy C. M., Marinho F. A., Santos L. O., Martins J. L. M., Santos A. L. S. and Branquinha M. H. (2006). Antileishmanial activity of MDL28170, a potent calpain inhibitor. International Journal of Antimicrobial Agents 28, 138–142. 16842979 10.1016/j.ijantimicag.2006.03.021 PMC7126437

[ref14] d'Avila-Levy C. M., Boucinha C. M., Kostygov A., Santos H. L. C., Morelli K. A., Grybchuk-Ieremenko A., Duval L., Votýpka J., Yurchenko V., Grellier P. and Lukes J. (2015). Exploring the environmental diversity of kinetoplastid flagellates in the high-throughput DNA sequencing era. Memórias do Instituto Oswaldo Cruz 110, 1–10. 10.1590/0074-02760150253 PMC4708014 26602872

[ref15] De Menezes J. P., Guedes C. E., Petersen A. L., Fraga D. B. and Veras P. S. (2015). Advances in development of new treatment for Leishmaniasis. BioMed Research International 2015, e815023. 10.1155/2015/815023 PMC4442256 26078965

[ref16] Desjeux P. (2001). The increase in risk factors for leishmaniasis worldwide. Transactions of the Royal Society of Tropical Medicine & Hygiene 95, 239–243. 11490989 10.1016/s0035-9203(01)90223-8

[ref17] Dey R., Bhattacharya J. and Datta S. C. (2006). Calcium-dependent proteolytic activity of a cysteine protease caldonopain is detected during *Leishmania* infection. Molecular and Cellular Biochemical 281, 27–33. 10.1007/s11010-006-0171-y 16328954

[ref18] Donkor I. O. (2011). Calpain inhibitors: a survey of compounds reported in the patent and scientific literature. Expert Opinion on Therapeutic Patents 21, 601–636. 21434837 10.1517/13543776.2011.568480

[ref19] Donkor I. O. (2015). An updated patent review of calpain inhibitors (2012–2014). Expert Opinion on Therapeutic Patents 25, 17–31. 25399719 10.1517/13543776.2014.982534

[ref20] Dorlo T. P., Balasegaram M., Beijnen J. H. and de Vries P. J. (2012). Miltefosine: a review of its pharmacology and therapeutic efficacy in the treatment of leishmaniasis. Journal of Antimicrobial Chemotherapy 67, 2576–2597. 22833634 10.1093/jac/dks275

[ref21] Ennes-Vidal V., Menna-Barreto R. F. S., Santos A. L. S., Branquinha M. H. and d'Avila-Levy C. M. (2010). Effects of the calpain inhibitor MDL28170 on the clinically relevant forms of *Trypanosoma cruziin vitro*. Journal of Antimicrobial Chemotherapy 65, e1395–1398. 10.1093/jac/dkq154 20457672

[ref22] Ennes-Vidal V., Menna-Barreto R. F. S., Santos A. L., Branquinha M. H. and d'Avila-Levy C. M. (2011). MDL28170, a calpain inhibitor, affects *Trypanosoma cruzi* metacyclogenesis, ultrastructure and attachment to *Rhodnius prolixus* midgut. Plos ONE 6, e18371. 21483751 10.1371/journal.pone.0018371 PMC3070728

[ref23] Ersfeld K., Barraclough H. and Gull K. (2005). Evolutionary reflationary relationships and protein domain architecture in an expanded calpain superfamily in kinetoplastid parasites. Journal of Molecular Evolution 61, 742–757. 16315106 10.1007/s00239-004-0272-8

[ref24] Ghofrani H. A., Osterloh I. H. and Grimminger F. (2006). Sildenafil: from angina to erectile dysfunction to pulmonary hypertension and beyond. Nature Reviews on Drug Discovery 5, 689–702. 16883306 10.1038/nrd2030 PMC7097805

[ref26] Hayes P., Varga V., Olego-Fernandez S., Sunter J., Ginger M. L. and Gull K. (2014). Modulation of a cytoskeletal calpain-like protein induces major transitions in trypanosome morphology. Journal of Cellular Biology 206, 377–384. 10.1083/jcb.201312067 PMC4121973 25092656

[ref27] Hook G., Hook V. Y. and Kindy M. (2007). Cysteine protease inhibitors reduce brain betaamyloid and beta-secretase activity *in vivo* and are potential Alzheimer's disease therapeutics. Biological Chemistry 388, 979–983. 17696783 10.1515/BC.2007.117

[ref28] Huang Y. and Wang K. K. (2001). The calpain family and human disease. Trends in Molecular Medicine 7, 355–362. 11516996 10.1016/s1471-4914(01)02049-4

[ref25] Jiao W., McDonald D. Q., Coxon J. M. and Parker E. J. (2010). Molecular modeling studies of peptide inhibitors highlight the importance of conformational prearrangement for inhibition of calpain. Biochemistry 49, 5533–5539. 20499928 10.1021/bi100048y

[ref29] Kawasaki H., Emori Y., Imajoh-Ohmi S., Minami Y. and Suzuki K. (1989). Identification and characterization of inhibitory sequences in four repeating domains of the endogenous inhibitor for calcium dependent protease. Journal of Biochemistry 106, 274–281. 2553682 10.1093/oxfordjournals.jbchem.a122844

[ref30] Kim S. H., Lee Y. H., Jung S. Y., Kim H. J., Jin C. and Lee Y. S. (2011). Synthesis of chromone carboxamide derivatives with antioxidative and calpain inhibitory properties. European Journal of Medicinal Chemistry 46, 1721–1728. 21397369 10.1016/j.ejmech.2011.02.025

[ref31] Kwok A. K. and Koenigbauer F. M. (2015). Incentives to repurpose existing drugs for orphan indications. ACS Medicinal Chemistry Letters 6, 828–830. 26288677 10.1021/acsmedchemlett.5b00276 PMC4538444

[ref32] Low K. E., Ler S., Chen K. J., Campbell R. L., Hickey J. L., Tan J., Scully C. C., Davies P. L., Yudin A. K. and Zaretsky S. (2016). Rational design of calpain inhibitors based on Calpastatin Peptidomimetics. Journal of Medical Chemistry 59, 5403–5415. 10.1021/acs.jmedchem.6b00267 27148623

[ref33] Machado-Silva A., Guimarães P. P., Tavares C. A. and Sinisterra R. D. (2015). New perspectives for leishmaniasis chemotherapy over current anti-leishmanial drugs: a patent landscape. Expert Opinion on Therapeutic Patents 25, 247–260. 25530084 10.1517/13543776.2014.993969

[ref34] Marinho F. A., Gonçalves K. C., Oliveira S. S. C., Gonçalves D. S., Matteolli F. P., Seabra S. H., Oliveira A. C. S., Bellio M., Oliveira S. S., Souto-Padrón T., d'Avila-Levy C. M., Santos A. L. S. and Branquinha M. H. (2014). The calpain inhibitor MDL28170 induces the expression of apoptotic markers in *Leishmania amazonensis* promastigotes. Plos ONE 9, e87659. 24498160 10.1371/journal.pone.0087659 PMC3909198

[ref35] Mehdi S. (1991). Cell-penetrating inhibitors of calpain. Trends in Biochemical Sciences 16, 150–153. 1877091 10.1016/0968-0004(91)90058-4

[ref36] Morton J. D., Lee H. Y., McDermott J. D., Robertson L. J., Bickerstaffe R., Jones M. A., Coxon J. M. and Abell A. D. (2013). A macrocyclic calpain inhibitor slows the development of inherited cortical cataracts in a sheep model. Investigative Ophthalmology and Visual Science 54, 389–395. 23211821 10.1167/iovs.12-11088

[ref37] Nwaka S. and Hudson A. (2006). Innovative lead discovery strategies for tropical diseases. Nature Reviews Drug Discovery 5, 941–955. 17080030 10.1038/nrd2144

[ref38] Olego-Fernandez S., Vaughan S., Shaw M. K., Gull K. and Ginger M. L. (2009). Cell morphogenesis of *Trypanosoma brucei* requires the paralogous, differentially expressed calpain-related proteins CAP5·5 and CAP5·5*V*. Protist 60, 576–590. 10.1016/j.protis.2009.05.003 19656721

[ref39] Otto H. H. and Schirmeister T. (1997). Cysteine proteases and their inhibitors. Chemistry Reviews 97, 133–172. 10.1021/cr950025u 11848867

[ref40] Saez M. E., Ramirez-Lorca R., Moron F. J. and Ruiz A. (2006). The therapeutic potential of the calpain family: new aspects. Drug Discovery Today 11, 917–923. 16997142 10.1016/j.drudis.2006.08.009

[ref41] Salotra P., Duncan R. C., Singh R., Subba Raju B. V., Sreenivas G. and Nakhasi H. L. (2006). Upregulation of surface proteins in *Leishmania donovani* isolated from patients of post kala-azar dermal leishmaniasis. Microbes and Infection 8, 637–644. 16469521 10.1016/j.micinf.2005.08.018

[ref42] Sangenito L. S., Ennes-Vidal V., Marinho F. A., Da Mota F. F., Santos A. L. S., Branquinha M. H. and d'Avila-Levy C. M. (2009). Arrested growth of *Trypanosoma cruzi* by the calpain inhibitor MDL28170 and detection of calpain homologues in epimastigote forms. Parasitology 136, 433–441. 19250597 10.1017/S0031182009005629

[ref43] Satoyashi E. (1992). Therapeutic trials on progressive muscular dystrophy. International Medicine 31, 841–846. 10.2169/internalmedicine.31.841 1450492

[ref44] Saxena A., Worthey E. A., Yan S., Leland A., Stuart K. D. and Myler P. J. (2003). Evaluation of differential gene expression in *Leishmania major* Friedlin procyclics and metacyclics using DNA microarray analysis. Molecular and Biochemical Parasitology 129, 103–114. 12798511 10.1016/s0166-6851(03)00100-2

[ref46] Sorimachi H., Hata S. and Ono Y. (2011). Calpain chronicle – an enzyme family under multidisciplinary characterization. Proceedings of the Japan Academy, Series B Physical Biological Sciences 87, 287–327. 10.2183/pjab.87.287 PMC3153876 21670566

[ref47] Tao M., Bihovsky R., Wells G. J. and Mallamo J. P. (1998). Novel peptidyl phosphorus derivatives as inhibitors of human calpain I. Journal of Medicinal Chemistry 41, 3912–3916. 9748367 10.1021/jm980325e

[ref48] Taylor G. J. and Wainwright P. (2005). Open label extension studies: research or marketing? British Medical Journal 331, 572–574. 16150772 10.1136/bmj.331.7516.572 PMC1200598

[ref49] Vergnes B., Gourbal B., Girard I., Sundar S., Drummelsmith J. and Ouellette M. (2007). A proteomics screen implicates HSP83 and a small kinetoplastid calpain-related protein in drug resistance in *Leishmania donovani* clinical field isolates by modulating drug-induced programmed cell death. Molecular and Cellular Proteomics 6, 88–101. 17050524 10.1074/mcp.M600319-MCP200

[ref50] Vicik R., Busemann M., Baumann K. and Schirmeister T. (2006). Inhibitors of cysteine proteases. Current Topics on Medicine Chemistry 6, 331–353. 10.2174/156802606776287081 16611146

[ref51] Wang K. K., Nath R., Posner A., Raser K. J., Buroker-Kilgore M., Hajimohammadreza I., Probert A. W. Jr., Marcoux F. W., Takano E., Hatanaka M., Maki M., Caner H., Collins J. L., Fergus A., Lee K. S., Lunney E. A., Hays S. J. and Yuen P. (1996). An alpha-mercaptoacrylic acid derivative is a selective nonpeptide cell-permeable calpain inhibitor and is neuroprotective. Proceedings of Natural Academic, Science USA 93, 6687–6692. 10.1073/pnas.93.13.6687 PMC39087 8692879

[ref52] Wilkinson S. R. and Kelly J. M. (2009). Trypanocidal drugs: mechanisms, resistance and new targets. Expert Reviews on Molecular Medicine 11, 1–24. 10.1017/S1462399409001252 19863838

[ref53] World Health Organization (2010). Control of the Leishmaniasis: Report of a Meeting of the WHO Expert Committee on the Control of Leishmaniasis. World Health Organization, Geneva, Switzerland.

[ref54] World Health Organization (2015). Neglected Diseases. World Health Organization, Geneva, Switzerland.

[ref55] Zhang Z., Huang Z., Dai H., Wei L., Sun S. and Gao F. (2015). Therapeutic efficacy of E-64-d, a selective calpain inhibitor, in experimental acute spinal cord injury. Biomed Research International 2015, 134242. 26240815 10.1155/2015/134242 PMC4512559

